# Nonviral delivery systems for antisense oligonucleotide therapeutics

**DOI:** 10.1186/s40824-022-00292-4

**Published:** 2022-09-30

**Authors:** Si Huang, Xin-Yan Hao, Yong-Jiang Li, Jun‑Yong Wu, Da-Xiong Xiang, Shilin Luo

**Affiliations:** 1 Department of Pharmacy, the Second Xiangya Hospital, Central South University, Changsha, 410011 People’s Republic of China; 2 Hunan Provincial Engineering Research Centre of Translational Medicine and Innovative Drug, Changsha, 410011 People’s Republic of China; 3 Institute of Clinical Pharmacy, Central South University, Changsha, China

**Keywords:** Antisense oligonucleotides, Nonviral delivery, Gene drugs, Nanoparticles

## Abstract

Antisense oligonucleotides (ASOs) are an important tool for the treatment of many genetic disorders. However, similar to other gene drugs, vectors are often required to protect them from degradation and clearance, and to accomplish their transport in vivo. Compared with viral vectors, artificial nonviral nanoparticles have a variety of design, synthesis, and formulation possibilities that can be selected to accomplish protection and delivery for specific applications, and they have served critical therapeutic purposes in animal model research and clinical applications, allowing safe and efficient gene delivery processes into the target cells. We believe that as new ASO drugs develop, the exploration for corresponding nonviral vectors is inevitable. Intensive development of nonviral vectors with improved delivery strategies based on specific targets can continue to expand the value of ASO therapeutic approaches. Here, we provide an overview of current nonviral delivery strategies, including ASOs modifications, action mechanisms, and multi-carrier methods, which aim to address the irreplaceable role of nonviral vectors in the progressive development of ASOs delivery.

## Introduction

In human disease treatment, antibody-based and conformation-corrected therapies that focus on the clearance of certain proteins associated with genetic diseases are being developed [1, 2], particularly because the bulk of therapeutic candidate target genes for genetic diseases are not the targets of the vast majority of small-molecule drugs. Accordingly, nucleic acid-based therapeutics have attracted the attention of researchers, and antisense technology is now beginning to deliver on its promise to treat diseases by targeting RNA [3, 4]. However, even though a wide selection of RNA sources, including precision duplex silencers RNA (siRNA), microRNAs, messenger RNA (mRNA), and RNA aptamers, are available for therapeutic use, the efficiency of the final conversion to reliable drugs is not ideal, and the current output of new drugs is limited [5, 6]. In contrast, short oligonucleotides that localize to the nucleus and provide a pathway for gene silencing by the RNase H pathway offer a more direct and reliable option.

**Fig. 1:**
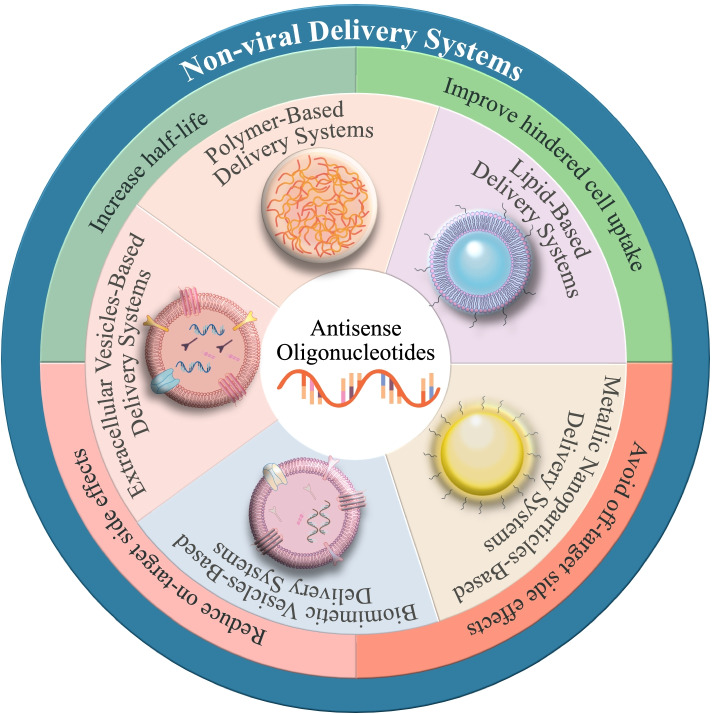
Overview of nonviral delivery systems for antisense oligonucleotide therapeutics

Antisense oligonucleotides (ASOs) are synthetic small single-stranded nucleic acid polymers (approximately 18 ~ 30 nucleotides) with diverse chemical properties that can be employed to regulate gene expression via various mechanisms. Unlike small-molecule drugs, antisense drugs work through Watson–Crick base pairing with the target RNA sequence [7]. This difference is believed to be the underlying reason for the excellent performance of ASOs in treating a variety of genetic disorders for which small-molecule drugs are not available [8]. Meanwhile, compared to RNAs which tolerate only limited modifications to remain RNA-Induced Silencing Complex (RISC) compatibility, one of their critical advantage is higher affinity, as the development of chemical modifications increases affinity, selectivity, and reduces toxicity due to off-target effects [9]. Since Fomiviren was approved by the FDA in 1998 for the treatment of retinitis caused by cytomegalovirus (CMV) infection in immunocompromised AIDS patients [10], several single-stranded antisense oligonucleotide (ASO) drugs belonging to multiple companies administered by four different routes have been approved for commercial use (Table 1), and even more ASOs with varying mechanisms of action and routes of administration are in preparation [4]. However, the extent of drug exploitation using vectors to deliver ASOs is still quite primitive, and this is one of the priorities for the future drug development of ASOs. This review provides an overview of the potentially valuable delivery strategies of ASOs based on nonviral vectors, the graphical overview is presented in Fig. 1.

**Table 1:** FDA-approved ASO therapeutics

Generic Name	Drug	Administration Route	Approval Year	Target	Indication	Applicant
VITRAVENE	Fomivirsen	Intravitreal injection	1998	Cytomegalovirus	Cytomegalovirus retinitis	IONIS Pharmaceuticals
MACUGEN	Pegaptanib	Intravitreal injection	2004	Vascular endothelial growth factor	Macular degeneration	EYETECH PHARMS
KYNAMRO	Mipromersen	SC injection	2013	Apo B-100 synthesis	Heterozygous familial hypercholesterolemia	KASTLE THERAPS LLC
SPINRAZA	Nusinersen	Intrathecal injection	2015	Mutations in chromosome 5q	Spinal Muscular Atrophy	BIOGEN IDEC
DEFITELIO	Defibrotide	IV infusion	2016	P38 mitogen-activated protein kinase	Sinusoidal obstructive syndrome	JAZZ PHARMS INC
EXONDYS 51	Eteplirsen	IV infusion	2016	Exon 51 of the dystrophin gene	Duchenne muscular dystrophy	SAREPTA THERAPS INC
TEGSEDI	Inotersen	SC injection	2018	Vascular endothelial growth factor	Macular degeneration	AKCEA THERAPY
VYONDYS 53	Golodirsen	IV infusion	2019	Exon 53 of the dystrophin gene	Duchenne muscular dystrophy	SAREPTA THERAPS INC
VILTEPSO	Viltolarsen	IV infusion	2020	Exon 53 of the dystrophin gene	Duchenne muscular dystrophy	NIPPON SHINYAKU
AMONDYS 45	Casimersen	IV infusion	2021	Exon 45 of the dystrophin gene	Duchenne muscular dystrophy	SAREPTA THERAPS INC

*SC *Subcutaneous, *IV *Intravenous

The data are extracted from the US-FDA official website: https://www.fda.gov/

### Modifications of antisense oligonucleotide structure

ASOs are synthetic oligonucleotides or oligonucleotide analogs that can be designed to bind to protein-coding RNAs as well as noncoding RNAs. They regulate RNA function through a variety of different mechanisms, depending on the types of chemical modifications, modification sites, and binding sites by which they target RNAs. Moreover, ASOs can be designed to regulate the processing of RNA molecules, including the regulation of RNA splicing and the selection of polyadenylation sites [11, 12], to disrupt the structure of RNAs used to inhibit the translation of proteins [13], and to promote the degradation of bound RNA by endogenous nucleases [14].

Due to hindered cell uptake [15, 16], off-target effects [17, 18], undesirable on-target effects [19], short half-life, immune clearance, and other disadvantages that free ASOs cannot avoid in vivo, researchers have proposed a variety of modifications to improve the stability and extend the half-life of ASOs [20, 21]. Phosphorothioate allows the nonbridging oxygen of the phosphate group in ASOs to be replaced by a sulfur group, resulting in the formation of a phosphorothioate bond, which is resistant to nuclease-based degradation [22]. In addition, the phosphorodiamidate morpholino modification increases the water solubility of ASOs [23], a peptide nucleic acid is an artificial mimic capable of self-assembly to form a backbone structure [24], and a locked nucleic acid is more commonly used today and can greatly increase the stability of ASOs [25], and 2′-*O*-methoxyethyl-(2′-*O*-MOE) and 2′-*O*-[2-(methylamino)-2-oxoethyl] improve the binding affinity of ASOs and provide resistance to enzymatic degradation [26].

### Mechanism of action of antisense nucleotides

**Fig. 2:**
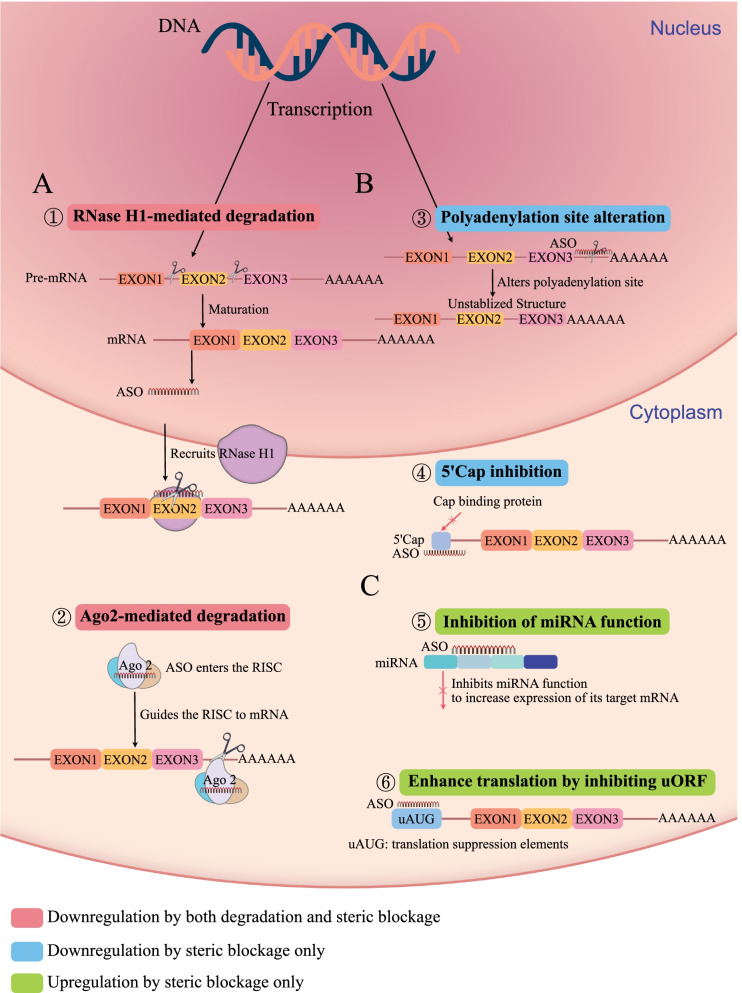
The main mechanisms of ASO regulate genes. **A** Downregulation mechanism of degradation and steric blockage simultaneously; ①. The ASO-mRNA double strands as a substrate recruit RNase H1, leading to degradation of the target transcript. ②. ASOs enter the RISC including a part in Ago 2, and become the guide strand. Then direct the RISC to mRNA. **B** Downregulation mechanism of steric blockage; ③. ASOs bind to pre-mRNA to alter polyadenylation position, and decrease mRNA stability and levels. ④. ASOs bind to the most 5ʹ region of mRNAs to avoid the binding of translation initiation factors, inhibiting translation. **C** Upregulation mechanism of steric blockage; ⑤. ASOs inhibit miRNA function to increase the expression of their target mRNA. ⑥. ASOs can enhance translation by inhibiting upstream open reading frames (uORFs), a translation suppression element

ASOs are theoretically designed to regulate the transfer of genetic information to proteins specifically, but the mechanisms by which ASOs induce biological effects are subtle and complex (Fig. 2). Based on the mechanism of action, two major classes of ASOs can be discerned: (a) Degradation by the RNase H and Argonaute 2 (the most widely be adopted two strategies) or some other elements (Fig. 2A), (b) Steric-blocker oligonucleotides, which physically block or inhibit the progression of splicing or translation mechanisms (Fig. 2B, C).

#### Regulation by degradation and steric blockage together

ASOs bind to target RNA to form a conjugate that recruits RNase H to degrade RNA for silencing [27, 28]. Degradation mediated by RNase H is the most stable and reliable mode of ASO action and is almost unaffected by the multiple modifications imposed on the ASOs themselves. Most FDA-approved ASO drugs work in this way. In addition, ASOs form double strands with the target RNA and then bind to the Argonaute 2 (Ago 2) enzyme to form the RISC. RISC moves to the complementary mRNA region where the Ago 2 enzyme breaks down the mRNA and exerts its gene silencing effect [29].

#### Regulation by steric blockage only

ASOs with steric blockage function are designed to bind to target transcripts with high affinity. Still, they do not induce degradation of the target transcripts due to their lack of RNase H recruitment capacity [30, 31]. This action is most commonly seen in phosphorodiamidate morpholino antisense-modified oligomers (PMOs), a class of antisense nucleic acid drugs that typically interfere with the expression of target genes by binding and spatially blocking the assembly of the translation machinery [32]. Unlike classical phosphorothioate oligonucleotides (PS-ODNs), PMOs do not induce RNase H activity, they bind to target RNA sequences and spatially block ribosome assembly or intron–exon splice junction sites, leading to translation arrest or splicing alteration. PMO-modified ASOs have different chemical properties from ASOs with other modifications: they are usually neutral rather than carrying charges [33]. Differences in chemically modified structures may lead not only to different mechanisms of action but also unique pharmacokinetics and biosafety of the ASO via steric blockade. As oligonucleotides that do not affect RNA integrity, steric-blocking ASOs have irreplaceable long-term potential and value in nucleic acid pharmaceuticals [34]. Other approaches include that ASOs modulate RNA function to attenuate or augment the translation of corresponding proteins in the cytoplasm. Moreover, ASOs can be designed to affect RNA splicing and polyadenylation site selection to regulate the processing of RNA molecules [35, 36]. In addition, ASOs designed to disrupt translation-suppressing RNA structures, block upstream AUG codons, or bind to microRNA can increase protein translation [13, 37].

### Promising delivery system for antisense oligonucleotides

#### Lipid-based delivery systems

**Fig. 3:**
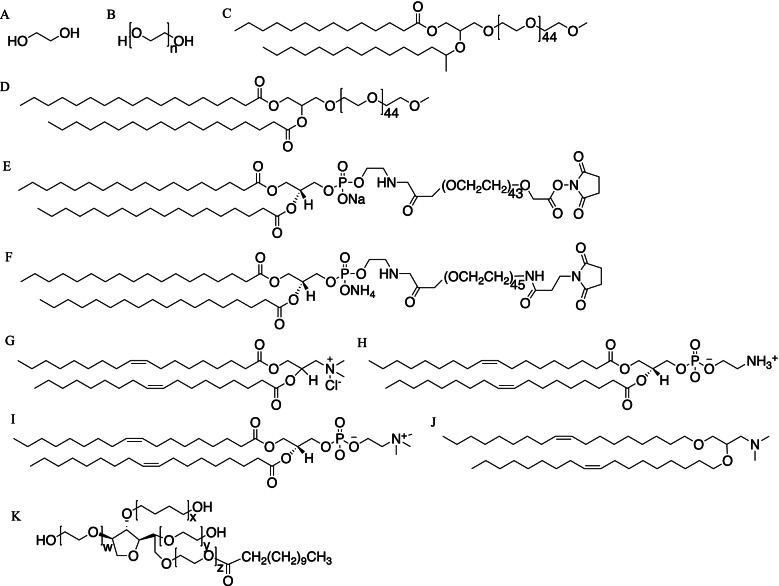
Chemical structure of Common PEG materials. (**A**) ethylene glycol, (**B**) linear polyethylene glycol, (**C**) DMG-PEG 2000, (**D**) DSG-PEG 2000, (**E**) DSPE-PEG(2000) carboxy NHS, (**F**) DSPE-PEG(2000) maleimide, (**G**) DOTAP(chloride salt), (**H**) DOPE, (**I**) DOPC, (**J**) DODMA, (**K**) branched PEG

To deliver ASOs to the target site by different routes of administration, nanocarriers of cationic polymers are usually preferred because of their ability to form polyelectrolyte complexes by facilitating ionic interactions between the positively charged functional groups and the negatively charged phosphate fraction [38]. The form of nucleic acids in nanocarriers is complex. ASOs can be encapsulated in the matrix of the nanocarrier or attached to the surface of the carrier by covalent or ionic bonding. Lipid-based nanoparticle (LNP) systems are one of the most promising colloidal nanocarriers for bioactive organic molecules. LNPs for the delivery of ASOs (LNPs-ASOs) typically consist of ionizable cationic lipids, phospholipids, polyethylene glycol (PEG) lipids, and cholesterol due to the negatively charged nature of nucleic acids [39–41]. PEG-series materials are structurally similar, but each has a specific structure and unique function (Fig. 3). The LNP-based delivery platform is appreciated as an advanced virus-free delivery system for ASOs for the treatment of a range of diseases [42]. Hitherto three LNP-based RNA drugs have been approved by the FDA, including two COVID-19 mRNA vaccines that play an irreplaceable role in preventing the spread of epidemics [43, 44].

LNPs have a suitable particle size (diameter range of 10–500 nm) combined with their own biocompatible and biodegradable lipids, which enables LNPs-ASOs to escape uptake by the mononuclear phagocyte system (MPS), subsequently prolonging the circulation time of LNPs-ASOs and allowing the particles to passively and efficiently target cells through an enhanced permeability and retention effect to release ASOs [45–47]. They also improve cell-to-ASO uptake by inducing lipid fusion between the membranes of LNPs and target cells during structure phase transitions [48–50], and help ASOs travel to target genes by promoting endosomal escape after cellular uptake [50, 51]. Examples of lipid-based delivery systems that effectively deliver ASOs are summarized in Table 2.

**Table 2:** Summary of the common lipid-based delivery system

Delivery systems	Administration routs	Targeted diseases	Nanocarriers components	Particle size	Key observations	Ref
Biodegradable lipid nanoparticles	Intravenous injection	PCSK9 in liver	306-O12B-3, DOPE, PEG, cholesterol, ASO	150–500 nm	ASO/LNP complexes reduce the total PCSK9 protein and serum cholesterol level with no hepatotoxicity or nephrotoxicity	[47]
Lipid nanoparticles	Intravenous injection	Metastatic renal cancer	DOTAP, soyPC, TPGS, folate-PEG-DSPE, ASO	108.6 ± 5.8 nm	Folate receptor-targeted lipid-albumin nanoparticles augment cell uptake rate and prolong the half-life of ASO	[48]
Lipid nanoparticles	Intravenous injection	Lung cancer	DODMA, egg PC, cholesterol, T7-PEG-DSPE, PEG-DMG, ASO	139.4 ± 7.6 nm	T7-conjugated CO-ASOs-LNPs exhibit excellent colloidal stability and produce superior antitumor activity	[49]
Lipid nanoparticles	Intravenous injection	Acute myelogenous leukemia	Cholesterol, DDAB, PEI, TPGS, Tf, DOTAP, DSPE-PEG2000-Mal, ASO	133.4 ± 7.6 nm	Transferrin-conjugated lipid nanoparticles augment cell uptake rate significantly	[50]
Lipid nanoparticles	Intravenous injection	Hepatocellular carcinoma	Neutral cytidinyl lipid, cystine skeleton cationic lipid, DSPE-PEG, ASO	139.0 ± 9.2 nm	Mix/CT102 nanoparticles exhibit a predominant accumulation capacity in liver tissue	[51]
Cationic liposomes	NA	Prostate cancer	DSPE-PEG2000-Mal, Cholesterol, DOTAP, Phosphatidylcholine, Trastuzumab, ASO	127 – 154 nm	ASO liposomes are more effective than free ASO to penetrate 2D and 3D spheroid models	[55]
Cationic elastic liposomes	Cutaneous administration	Atopic dermatitis	DOTAP, Sodium cholate, ASO	Over 200 nm	IL-13 ASO/cationic elastic liposomes dramatically suppress IL-13 production (by up to 70% of free ASO)	[56]
Cationic liposomes	Injection	*Candida albicans* infection	DOTAP, DOPC; DOPE, MO, ASO	40 – 80 nm	DOTAP-based lipoplexes inhibit *Candida albicans* filamentation up to 60% after 72 h	[57]
Hydrogel liposomes	Subcutaneous administration	NA	Lipid-oligonucleotides, hydrogels, ASO	14 nm	Hydrogel-based liposomes prolong ASO release and enhance its stability	[58]
Lipid nanoparticles	Peritumoural injection	Subcutaneous tumor	Cytidinyl lipid, cationic lipid, ASO	236 ± 7.9 nm	Cytidinyl-lipid combined with a cationic lipid exhibits high encapsulation efficiency for ASO	[59]
Lipid nanoparticles	Intravenous injection	Acute myelogenous leukemia	DOTAP, DOPE, TPGS, Cholesterol, DOC, PEI, ASO	93 ± 18 nm	CD33-targeted lipid nanoparticles show a 15-fold reduction in the IC50 of an antileukemic drug	[60]

DOTAP, cationic 1,2-dioleoyl-3-trimethylammonium-propane; DOPC, 1,2-dioleoyl-sn-glycero-3-phosphocholine; DOPE, 1,2-dioleoyl-*sn*-glycero-3-phosphoethanolamine; MO, monoolein; SoyPC, L-α-phosphatidylcholine; TPGS, DL-α-Tocopherol methoxy-polyethylene glycol succinate; DODMA, 1,2-Dioleyloxy-3-dimethylaminopropane; egg PC, egg l-α-phosphatidylcholine; PEG-DMG, 1,2-Dimyristoyl-rac-glycero-3-methylpolyoxyethylene; DOC, deoxycholate; AML, acute myeloid leukemia; DDAB, didecyldimethylammonium bromide; Tf, human holo-transferrin; DSPE-PEG2000-Mal, 1,2-distearoyl-sn-glycero-3-phosphoethanolamine-N-[maleimide (polyethylene glycol)-2000]; PEI, polyethylenimine

Several prior studies have reported LNPs to be potentially exploitable. Although some studies have not used LNPs as traditional formulations of nucleic acid drugs, the addition of lipid components alone reduces positive charge toxicity and can significantly improve biocompatibility [47]. However, the optimal composition of LNPs applicable to ASOs will vary, as will the key to successful delivery mechanisms[52, 53]. *Hiroki* et al. initially hypothesized that the optimal composition of ssPalmO-Phe/Chol for siRNA delivery could be applied to ASOs, but it was revealed that LNP_ssPalmO-Phe_ containing ASOs are highly unstable and susceptible to aggregation [54]. With an improved lipid composition and lipid/ASO ratio, an LNP system that could efficiently transport ASOs was obtained.

#### *Liposomes*

Liposomes are used in the pharmaceutical and cosmetic industries to transport a wide range of molecules. They are spherical vesicles composed of phospholipids and sterols, usually in the size range of less than 500 nm [61]. Liposomes are classified into several types based on the addition of PEG and ligands [62, 63]. PEG is arguably the most critical component of liposomes, which limits the adsorption of serum proteins and effectively prolongs blood circulation time [64]. While PEG has a recognized effect of improving the pharmacokinetic properties of nucleic acids, it is posing other challenges. The first is the hindrance of tissue penetration, cellular uptake, and endosomal escape behavior [65, 66]. The second is the repeated use of polyethylene glycol-modified liposomes, which inevitably leads to faster serum clearance and severely compromises subsequent therapeutic efficacy [67–69]. Despite the apparent disadvantages of PEG, there is still a lack of proven and reliable substitutes.

Liposomes are potentially more enriched in the liver and spleen than other carriers, so it is essential either to develop different types of liposomes to counteract this property or to take advantage of this property to deliver ASOs that are expected to work in these organs [70, 71]. The lipid component of the liposome stabilizes proteins on the surface, making it more advantageous to apply protein modifications. *Guan* et al. functionalized liposomes with a tumor-homing and -penetrating peptide, iRGD, as a carrier of an ASO against androgen receptor (AR) for prostate cancer treatment, and these iRGD-liposomes markedly improved the ASO efficacy in suppressing the growth of tumor [72]. The modification of liposomes with targeting antibodies improves the affinity of liposomes for cancer cells and optimizes the intratumoral penetration of ASOs [73].

#### Polymer-based delivery systems

Polymers have been one of the most widely used drug delivery systems since being discovered. In addition to proteins and small molecules, polymer-drug systems are also essential for the delivery of nucleic acid drugs [74, 75]. The classification of polymer systems is also highly complex with numerous categories according to the structural differences of the components. Examples of polymer-based ASO delivery systems are summarized in Table 3. The unique advantage of the polymer system is its stability. Because polymeric materials mostly have rigid shapes, polymeric nanoparticles can retain the ASOs carried in the central cavity of the nanoparticle even after a variety of operations such as long storage, lyophilization, concentration, and so on [76–78]. Here, we refer to the traditional classification method and divide them into four categories: early linear polycations, dendrimers, polymeric nanoparticles, and natural polymers [74]. One study claims that cationic micelles offer both the properties of cationic polymers and the benefits of micelles, with the added benefit of reduced toxicities [79]. The molecular structure of several classic materials and the structure of the carriers obtained by assembling them were shown in Fig. 3.

**Table 3:** Summary of common polymer-based delivery systems

Delivery Systems	Administration Route	Target	Nanocarriers components	Particle size	Key observations	Ref
Glucose-Coated polymeric nanocarrier	Intravenous injection	Brain	Glu-PEG-PLL, MeO-PEG-PLL	42–45 nm	Glucose-modified polymeric nanocarriers enable noninvasive ASO administration to the brain	[80]
Polyamide nanocarrier	Injection	*Candida albicans*	Porous poly(γ-butyrolactam), poly(ε-caprolactam), ASO	NA	Polyamide nanocarriers deliver ASO with entrapment or immobilization strategies	[81]
Polyethylenimine-based lipid nanoparticles	NA	Breast cancer	PEI, PC, Octaarginine, Palmitic acid, ASO	276.87 ± 5.63 nm	Lp-PPRP deliver ASOs with lower cytotoxic and higher transfection efficiency	[82]
Chitosan microparticles	NA	NA	Chitosan, ASO	200 μm	Chitosan microparticles maintain the stability of ASO in plasma	[83]
Core–shell nanoparticles	Intravenous injection	Lung cancer	α-tocopherol succinate, poly (lactic acid)-g-poly(ethylene glycol), ASO	220 ± 0.02 nm	ASO-modified nanoparticles exhibited good cellular internalization, cytotoxicity, and apoptotic and necrotic effects	[84]
Polyethylene glycol nanoparticles	Intravenous injection	Pancreatic cancer	Polyethylene glycol, polyethyleneimine, gemcitabine, ASO	40–120 nm	ASO accumulates at the tumor site significantly	[85]
Nanoparticles	Peritumoural injection	Drug-resistant bacteria	Zeolite imidazole framework-8, glucose oxidase, horseradish peroxidase, ASO	About 410 nm	Biomineralized nanoparticles with ASO achieved a high-efficiency treatment of MRSA infection	[86]
Dendrimer nanocomplex	Peritumoural injection	Skin tumor	PAMAM, ASO	80–150 nm	ASO-dendrimer complex causes significant apoptosis in skin tumor	[87]

*PEI *Polyethyleneimine, *PC *Palmitoyl chloride, *SPIO *Superparamagnetic iron oxide, *PAMAM *Polyamidoamine

#### *Early linear polycations*

Linear cationic compounds have long been shown to be effective in delivering nucleic acids. In the 1960s, these polycationic derivatives of dextran were shown to enhance the transfection of viral RNA and DNA [88, 89]. The advantages of Deae-dextran are chemical simplicity, reproducibility, and low cost, but the disadvantages are low transfection efficiency, cytotoxicity, and inhibition of cell growth in vitro, which limit its use in vivo. The discovery of linear polycations was of epoch-making significance, but linear polycations were soon replaced by dendritic polycations with complex and variable structures due to the insurmountable defects mentioned above.

#### *Micelles*

Micelles, self-assembled from block copolymers, have a unique core–shell structure with a size distribution in the range of 10–100 nm [90–92]. Although most available cationic polymers can coalesce DNA, they interact weakly with DNA. Thus, the polymers formed in physiological fluids, which contain serum components and salts that tend to break down these complexes, are not very stable. Therefore, they are not the best materials to form micelles for the delivery of ASOs [93, 94]. Furthermore, the synthesis of high molecular weight cationic polymers (e.g. dendrimers) is usually labor intensive and costly, greatly hindering their biomedical applications [95]. The self-assembly of amphiphilic polymers into micelles makes them excellent gene carriers. Amphiphilic cationic polymers such as polylysine (PLL) [96], PEI [97, 98], polyamidoamine (PAMAM) [99], and polydimethylaminoethyl methacrylate (PDMAEMA) [100, 101] are commonly used to construct cationic micelles [102, 103].

#### *Dendrimers*

**Fig. 4:**
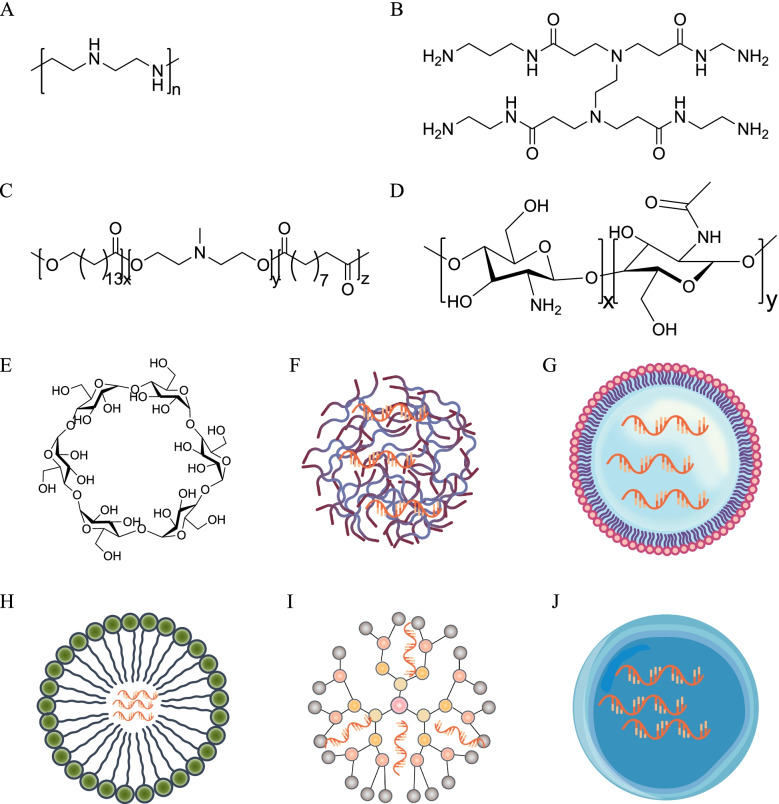
Chemical structure of polymer materials and schematic representation of different 11 particle forms. (**A**) PEI, (**B**) PAMAM, (**C**) PACEs, (**D**) chitosan, (**E**) α-cyclodextrins, (**F**) polyplexes, (**G**) nanocapsules, (**H**) micelles, (**I**) dendrimers, (**J**) nanoparticles

Synthetic polycations such as PEI and PAMAM dendrimers, and some other polycations, such as poly(amine-co-ester) (PACE) are included in this category [104–106] (Fig. 4A-C). Due to their extrinsic positive charge, ASO nanocarriers based on electrostatic adsorption are usually prone to nucleic acid leakage through the formation of polyelectrolyte aggregates and induce excessive positive charge-related cytotoxicity and non-specific interactions with serum or plasma proteins, but most of them have been used successively to deliver siRNA and mRNA with good results, however, only a few of which have been used to attempt the delivery of ASOs.

Marcel developed a nanoparticle-based delivery system for ASOs targeting the antibiotic resistance of methicillin-resistant *Staphylococcus aureus* (MRSA): the system was prepared by the sequential modification of gold nanoparticles with PEI and maintained antibacterial ability with reduced low cytotoxicity [107]. Yoshida succeeded in solving the problem of poor intracellular uptake by target cells by using superparamagnetic iron oxide (SPIO) nanoparticles coated with PEI as a delivery vehicle for ASOs [108].

PAMAM is another cationic dendrimer used to deliver ASOs. A co-delivery system is based on a cationic dendrimer core that encapsulates fluorouracil and oligonucleotides within a hydrophobic lumen, modified with hyaluronic acid and cell-penetrating peptides. The codelivery complex showed efficient cellular uptake and consequently improved intracellular distribution and enhanced cytotoxicity on cells [87, 109].

#### *Polymeric nanoparticles*

Polymeric nanoparticles, due to their tunable architecture (10–1000 nm), nontoxicity, biocompatibility, and controlled drug release are promising options for targeted drug delivery platforms [80, 110]. Widely used biodegradable synthetic polymers include poly(lactic acid) (PLA), poly(glycolic acid) (PGA), and copolymers such as poly(lactic acid-glycolic acid) (PLGA) [111]. PLGAs have been approved by the FDA for certain transport applications. These materials are difficult to use for nucleic acid delivery because unlike cationic polymers, they cannot rely on charge dominance to hold the nucleic acid [112, 113]. Therefore, PLGA is often used in conjunction with the cationic polymers PEI and PAMAM. The advantage of this is that not only does it rely on the electrical charge to achieve a higher nucleic acid loading, but also the addition of PLGA results in lower surface charge compared to the cationic polymer particles in isolation, resulting in lower toxicity and a lower rate of removal, which facilitates the sustained release of cargo [114]. In addition to dendrimers, other cationic polymers have been used in blends with PLGA, such as Poly-beta-amino-ester (PBAE), which has positively charged groups that can interact with nucleic acids and are simple to synthesize and more readily degraded in vivo [115, 116]. In the blend of PBAE and PLGA, cytotoxicity decreased with the ratio of PBAE to PLGA [117]. PACEs are another commonly used class of cationic polymers with the unique advantage of lower toxicity that results from lower charge density [118, 119]. PACE is more closely associated with plants than the compounds mentioned above, and a variety of PACEs are now derived indirectly or directly from plant-derived components [120]. *Cui* loaded solid PACE nanoparticles (PACE-NPs) with oligonucleotides designed to knockdown Nogo-B, a protein that has been implicated in the progression of alcoholic liver disease and liver fibrosis, and demonstrates that PACE-NPs can effectively deliver oligonucleotides therapeutics to the liver to mediate protein knockdown in vivo [121].

The synthesis reactions of polymers are quite mature, so chemical precision and flexibility in designing synthetic strategies are considerable; accordingly, a highly functionalized nucleic acid polymer (HFNAP) library is usually designed as needed, and the target compounds are screened by parallel experiments [122, 123]. In addition to the ability to design synthesis methods based on the desired chemical structure, hydrophilicity/hydrophobicity, charge density, and functional domains and structure, it is even possible to select suitable polymers based on the delivered nucleic acid sequence [124, 125].

#### *Natural polymer-based delivery systems*

Naturally derived structural proteins and polysaccharides, such as cationic collagen derivatives, cyclodextrins (CDs), and chitosan, have been developed as gene carriers [126–129]. Collagen, an important component of bone tissue, has a complex structure that makes it available as an artificial scaffold material with an innate drug retentive function. Natural collagen exists in two forms: as a swollen hydrogel and as sparse fibers in lattice-like tissues [130]. When targeting RNA delivery to bone-related cells, collagen should be the first candidate considered as a carrier material that provides good and stable sustained release [131]. In addition to scaffolds, collagen can be used in conjunction with polymers and lipids, as with other organic materials, to make nano preparation suitable for local injection with a slow-release, low systemic circulating drug concentration, and excellent specificity.

Due to the poor specificity, low stability, and low permeability of ASOs through cell membranes, an effective nucleic acid carrier system for most studies usually requires cationic materials. Chitosan is a strong candidate due to its cationic properties, biodegradability, and excellent biocompatibility [132]. Chitosan is a linear polymer formed by α (1 → 4)-linked-2-amino-2-deoxy-β-d-glucopyranose [133]. Various functional groups or molecules can be affixed with chitosan to guarantee the desired function with the nanocarrier system [134]. *Kolonko* et al. developed a nonviral delivery system based on the natural aminopolysaccharide chitosan (CS) for the transport of ASOs against ENaC to specifically address Na + hyperabsorption and confirmed the successful uptake of the nanocomplex by human airway epithelial cells, demonstrating the possibility of targeted transport of ASOs with chitosan [135].

#### Extracellular vesicle-based systems

**Fig. 5:**
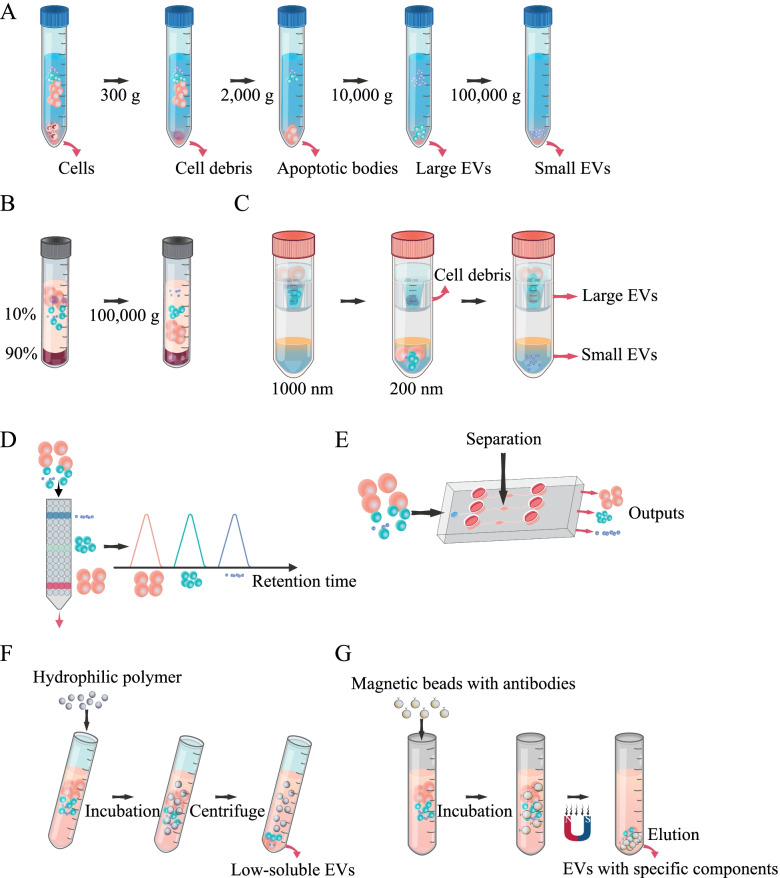
Schematic diagram of various schemes for collecting extracellular vesicles. **A** The cell supernatant was separated by repeated multiple ultracentrifugations to obtain EVs. **B** The supernatant was subjected to sucrose density gradient centrifugation, and EVs with different particle sizes were distributed in different concentrations of sucrose solution. **C** The separation of exosomes by rotary ultrafiltration technology is based on the principle that the pore size of the ultrafiltration membrane allows and intercepts substances of different relative molecular masses, filtering solvents and some small molecules to the other side of the membrane while retaining substances with high relative molecular mass that are larger than the membrane pore size on the ultrafiltration membrane, thus achieving separation. **D** Exclusion chromatography separates EVs of different particle sizes due to their different peak emergence times after passing through the column. **E** The microfluidic technique achieves exosome isolation, concentration, and analysis. **F** Particles of different sizes are subjected to differentially sized acoustic radiation and viscous forces in the microfluidic acoustic field. Under the combined effect of acoustic radiation and viscous force, particles of different sizes move to different exits, thus achieving separation. **G** Highly hydrophilic polymers interact with water molecules around exosomes to form a hydrophobic microenvironment, which leads to exosome precipitation. **H** EVs have specific markers on their surface and are adsorbed onto magnetic beads encapsulated with anti-marker antibodies that bind to exosome vesicles after incubation

The naming of extracellular vesicles is extremely chaotic. Extracellular vesicles (EVs) are vesicles that are released from cells into the extracellular space, and can be subdivided into microvesicles (100 nm to 500 nm in diameter) or exosomes (30 nm to 100 nm in diameter) by their specific diameter [136, 137], EVs are vesicles that carry nucleic acids and proteins that are secreted by almost all cells into the extracellular fluid and body fluids such as blood, urine, tears, and milk. Because of the propensity of EVs to transfer to recipient cells and the compositional advantages in biocompatibility, they are naturally used as vehicles for the delivery of nucleic acids. Seven classes of exosome isolation strategies have been reported, including stepwise ultracentrifugation (Fig. 5A), gradient density ultracentrifugation (Fig. 5B), ultrafiltration (Fig. 5C), size-exclusion chromatography (Fig. 5D), microfluidic techniques (Fig. 5E), polymer precipitation (Fig. 5F), and immunoaffinity capture (Fig. 5G), each of which has unique advantages and disadvantages. EVs are potent cell-derived nanovesicles that can mediate intracellular communication to achieve nondestructive and efficient delivery.

The ASO is usually loaded into the exosome by electroporation [138, 139]. The drug loading rate depends on the specific experimental conditions and the type of vesicles, but in general, it is relatively low compared to that of artificial carriers. Although more studies are reporting that EVs carrying ASOs can achieve good therapeutic effects, the mode of administration of the studies appears to be limited to injection, and in one study where ASOs was administered by oral delivery of bovine extracellular vesicles, no significant decrease in target gene expression was seen in vivo [140]. In addition to EVs produced by normal cellular secretion, EVs obtained by various artificial intervention methods have also been used to carry ASOs. Compared to exosomes, apoptotic bodies (ABs) can be produced with much higher efficiency [141].

#### Biomimetic vesicle-based systems

**Fig. 6:**
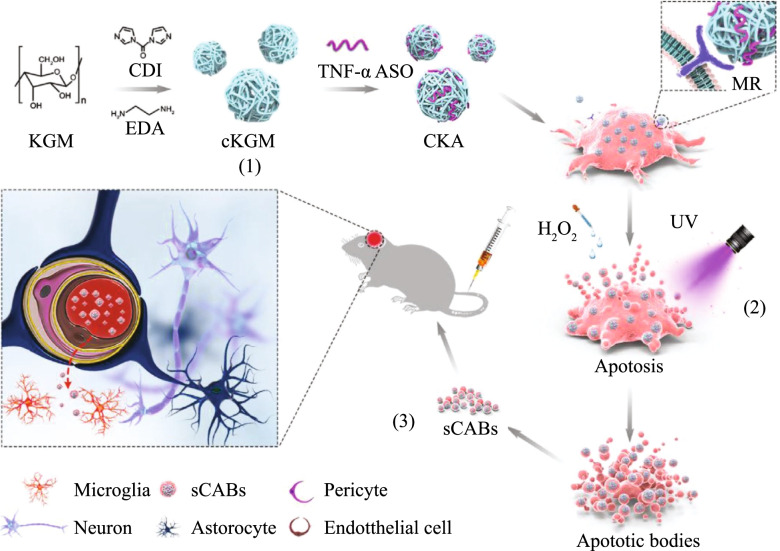
The ASOs bound to the cationic material can be made available for cell uptake, and then apoptotic vesicles containing ASOs can be directly produced by cell induction of cells after uptake of the nucleic acid drug. Schematic diagram of the protocol for producing small apoptotic bodies and delivering ASOs into the brain. (Reprinted with permission from Ref [142]. Copyright © 2021 The Authors. Advanced Science published by Wiley‐VCH GmbH)

**Fig. 7:**
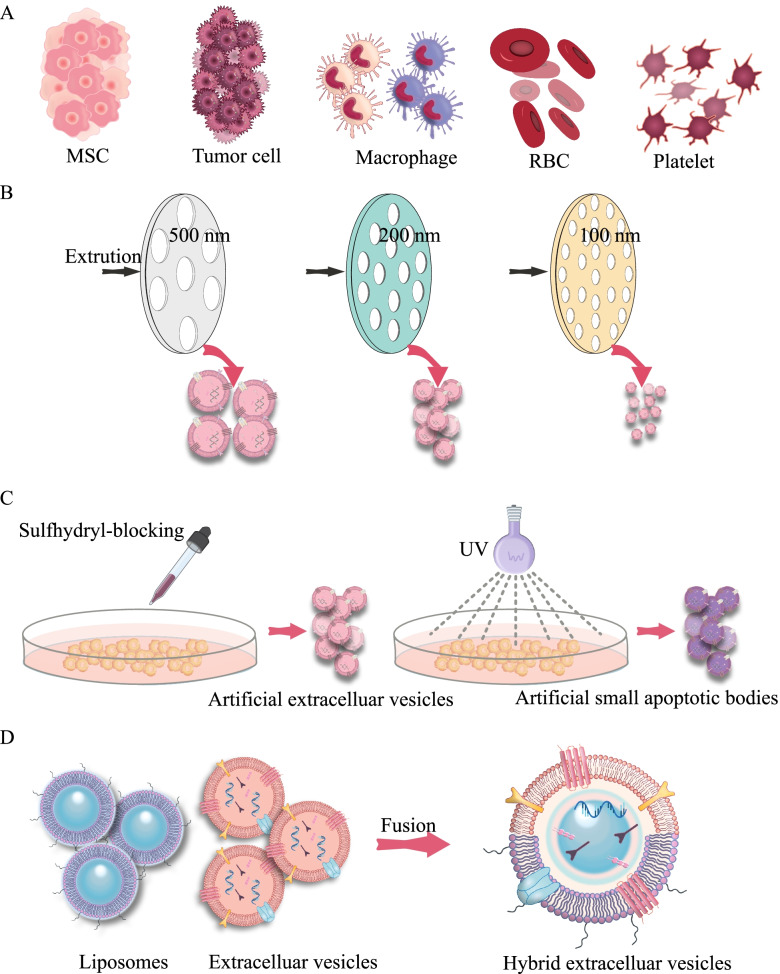
Biomimetic carriers for ASO delivery. **A** Components from a variety of cells and body fluids have been used to prepare biomimetic nanoparticles. **B** Cells can be forced to pass through membrane pores to form biomimetic nanoparticles. **C** Sulfhydryl-blocking can lead to the release of small biomimetic nanoparticles from the cell; UV light can induce apoptosis and produce small apoptotic bodies. **D** Isolated natural EVs and liposome nanoparticles can be fused into hybrid EVs

While delivery systems for artificial materials are not immune to compatibility and clearance problems, biological vesicles represented by exosomes are not immune to another challenge: low nucleic acid loading rates and the potential safety threat of carrying their own nucleic acids. The loading of nucleic acids onto vectors is low in efficiency and their functional activity may be compromised [143]. It was found that the number of nucleic acids loaded into EVs was limited [144]. Therefore, one study pretransfected ASOs into cells and then produced ASO-rich apoptotic vesicles by inducing apoptosis, and good genetic suppression was achieved with these apoptotic vesicles (Fig. 6) [142]. Currently, cell and organelle membranes derived from various cell types have been developed as carriers to deliver ASOs (Fig. 7A). Moreover, we summarized other methods in nanofabrication of artificial EVs, including extrusion (Fig. 7B), promoting secretion (Fig. 7C), and fusion (Fig. 7D). Bionic carriers are a new type of drug delivery system that has been rapidly developed in recent years and has the potential to solve many long-existing challenges at once. Biomimetic vectors are usually composed of endocytic, protein, organelle, microbial or viral structures with artificial nanoparticle materials or individually [145–148]. The commercialization of biomimetic drug delivery systems presents quality control and ethical issues, but such drug delivery systems are promising in terms of therapeutic efficacy.

#### Metallic nanoparticles systems

**Fig. 8:**
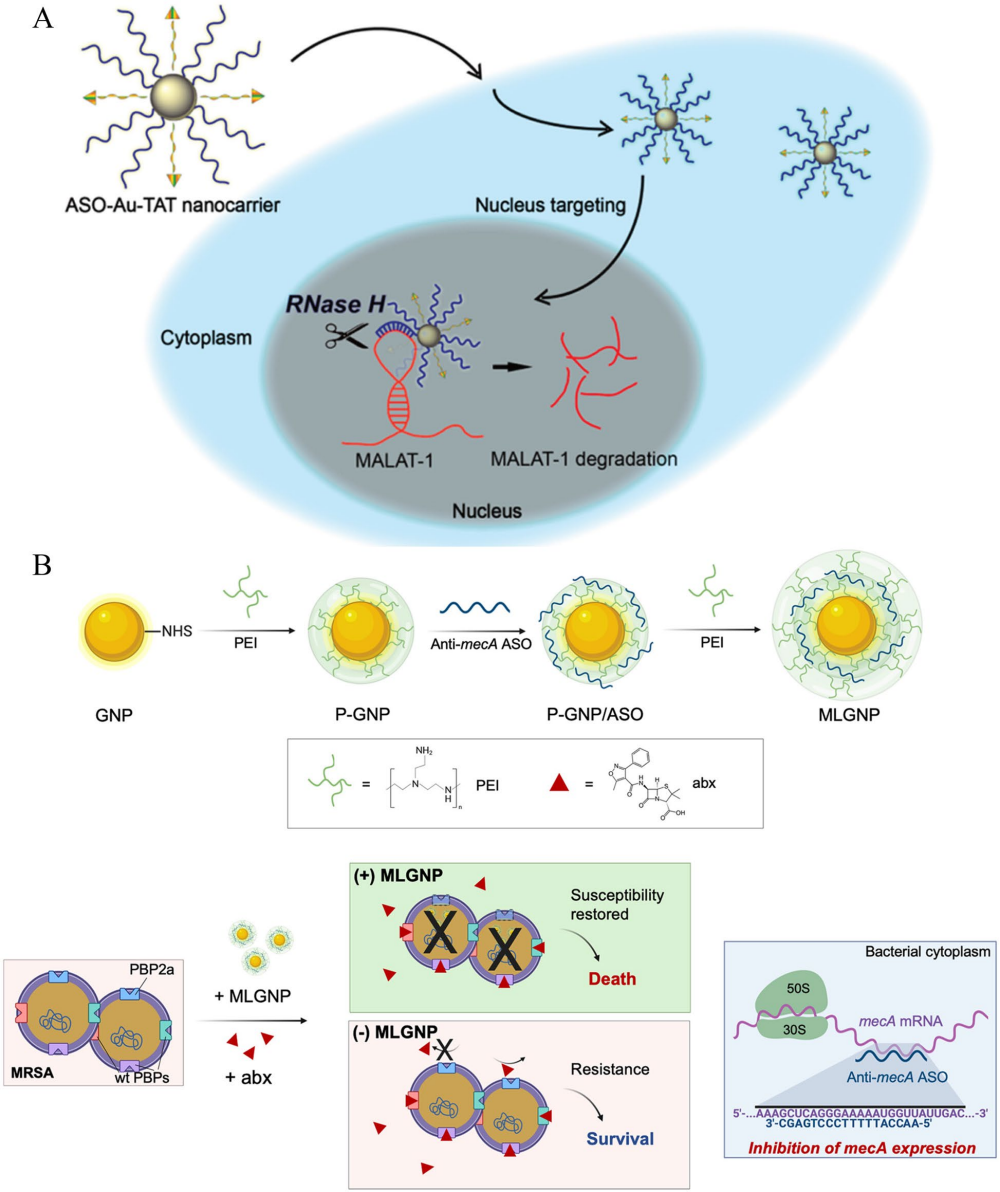
Schematic illustration of two gold nanoparticle systems. **(A**) Schematic illustration of nucleus-targeting by ASO-Au-TAT nanocarrier. (**B**) Schematic illustration for preparation of MLGNPs delivering ASO targeting antibiotic resistance and its application for combinatorial treatment of MRSA infections. (Fig. 8A reprinted with permission from Ref [154]. Copyright © 2019, American Chemical Society. Figure 8B reprinted with permission from Ref [107]. Copyright © 2021, Elsevier B.V.)

Metal nanoparticles are widely used and recognized in the fields of biotechnology and bioengineering [149]. Currently, metal nanoparticles and conjugates of ligands, drugs, antibodies, peptides, and nucleic acids have been used for targeted drug delivery, diagnostics, and imaging. Among the most studied are gold, silver, and platinum nanoparticles [150, 151]. Gold nanoparticles are the most widely studied and stable, with negligible toxicity and good imaging in vivo [152]. *Anna Graczyk *et al. invented a conjugate of gold nanoparticles and structural RNA that was successfully used as a tool for gene expression regulation successfully [153]. *Gong *et al. constructed MALAT1-specific ASO and nucleus-targeting TAT peptide cofunctionalized Au nanoparticles, namely, ASO-Au-TAT NPs, which stabilized fragile ASOs, enhanced nuclear internalization, and exhibited good biocompatibility [154] (Fig. 8A). A multi-layer coated gold nanoparticles (MLGNPs) delivering antisense oligonucleotides (ASOs) were shown to be efficiently internalized into various types of Gram-positive bacteria and may use with conventional antibiotics [107] (Fig. 8B). The biocompatibility of metal nanoparticles and the functionalization of unrestricted nucleic acid structures offer a wide range of potential applications. They have emerged as an outstandingly promising solution for ASO delivery and personalized nanomedicine in the future.

#### Potent supporters: specific condition-sensitive materials

**Fig. 9:**
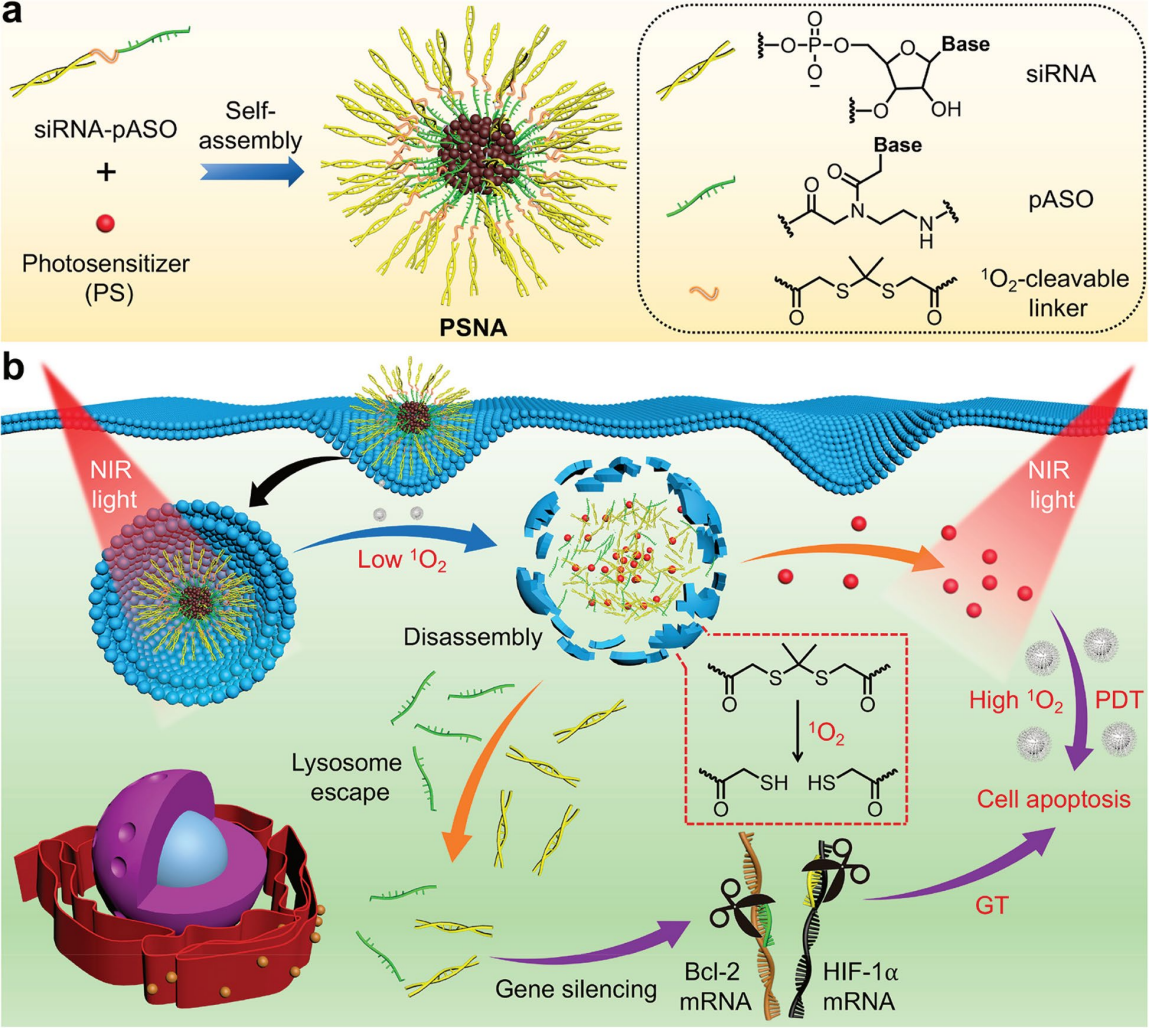
Design of photolabile spherical nucleic acid (PSNA). (**a**) Illustration of the preparation of PSNA. (**b**) Schematic representation of the use of PSNA to deliver siRNA, pASO, and PS for combination cancer therapy. (Reprinted with permission from Ref [161]. Copyright © 2021, American Chemical Society.)

Sensitive materials have a rich history of use in the treatment of infections and tumors with superior results [155, 156]. Since the approval of sodium polyphotodynamic therapy as the first photosensitizer (PS) for the treatment of bladder cancer in 1993, photodynamic therapy (PDT) has been widely used in antitumor and anti-infection therapy [157]. Photosensitizers have been tried in many nanoparticle systems [158, 159], and surprising results have been reported. In 2012, a study attempted to address the headache-inducing off-target effects of nucleic acid drugs by using a photosensitizer to trap the RNA carrier/siRNA complex completely within the endosome [160]. A near-infrared (NIR) photocontrolled self-delivery of ASO was designed to suppress hypoxia inducible factor-1α (HIF-1α) and B-cell lymphoma 2 (Bcl-2) for gene therapy [161] (Fig. 9). This precise, light-dependent control will open new possibilities for cellular and molecular biology and therapy.

Thermosensitive materials can also help retain ASO in local tissues without a serious off-target effect [162]. As confirmed in a study, a type of PLGA-PEG-PLGA thermosensitive hydrogel can increase the residence time of RNA nanoparticles in the eye to prolong the duration of action time of subconjunctival administration [163]. pH-sensitive hydrophobic fragments have been shown to promote the efficiency of oligonucleotide drug delivery by amphiphilic polycationic carriers [164]. The development of multifunctional drug nanoparticles that combine oligonucleotide drugs with different release mechanisms including thermosensitive, photosensitized, ultrasound-responsive [165], redox-responsive [166], and magnetic-responsive material [167], may be useful for specific applications. Sensitive materials can be used in a variety of diseases, perform well in clinical evaluation and assessment, and offer exciting possibilities in moving from the laboratory to real-world use.

### Challenges facing ASOs delivery

ASOs are readily degraded by nucleases in body fluids and are enriched in metabolic organs such as the liver and kidney, where they are rapidly cleared, and the half-life of unmodified and unencapsulated ASOs is usually less than 10 min [168–170]. In addition, the lack of targeting and the off-target effects of ASOs may lead to serious side effects and consequences, limiting both the dose administered and the therapeutic effect [171–174]. The negative electrical properties and high molecular weight of ASOs are also important factors, and the chemical structure of single nucleotide chains prevents their active uptake by the cell in any form and therefore makes it difficult to cross the cell membrane to enter the cell [173, 175]. Internalized ASOs are transported out of the body by the endosomal and acidic lysosomal microenvironments, making it difficult for them to enter the nucleus to act on target gene sites [176].

Although ASO drugs continue to come to market, safety has been a stubborn factor preventing them from expanding their impact [177]. An investigator from US-FDA noted that adverse reactions among preclinical and clinical study volunteers tended to occur in those who took ASO drugs intravenously, possibly because the systemic exposure to ASOs via this route was much higher than in other local ways [178]. Another challenge ASOs once got entangled with but now have tackled is that current approved ASO are limited to treating genetic diseases by causing alternative splicing in patients with loss-of-function mutations. Since the validation of new mechanisms of action enhances the versatility of antisense technology [179–181].

The human system is way more complex than the in vitro culture systems or even model animals [182, 183]; delivery systems in the human body are not yet fully understood so far, so no surprises that many ASO delivery systems research cases perform well in vitro but poorly in clinical studies [184, 185]. And the accuracy and affordability of synthetic polymers, as well as the safety and stability of biological components, are challenges. Carrier systems demand not only safety, low cost, and ease of manufacturing, but also controllability and stability to advance further toward the clinic [186–188]. Many delivery system formulations that perform well in laboratory studies may not always accomplish equally well under the harsh storage conditions of real-world applications, which hinders delivery systems from providing value.

## Conclusions

At this time, no ASO drugs using drug delivery systems have been approved by the FDA for marketing, and there will be no substitute for ASO therapeutic technologies for rare diseases for a significant period. It is foreseeable that the emergence of ASO drugs delivered by carriers is inevitable, but the timing depends on innovations in delivery systems. This will require breakthroughs in the development of materials, evaluation systems, synthesis methods, ethical safety, and many other aspects. Given the rapid progress in this field, nonviral delivery systems will certainly play an irreplaceable role in the progressive development of gene therapy.

## Data Availability

Not applicable.

## Abbreviations

Abs: Apoptotic bodies
Ago 2: Argonaute 2
AML: Acute myeloid leukemia
AR: Androgen receptor
ASO: Antisense oligonucleotide
CDs: Cyclodextrins
CMV: Cytomegalovirus
CS: Aminopolysaccharide chitosan
DDAB: Didecyldimethylammonium bromide
DOC: Deoxycholate
DODMA: 1,2-Dioleyloxy-3-dimethylaminopropane
DOPC: 1,2-Dioleoyl-sn-glycero-3-phosphocholine
DOPE: 1,2-Dioleoyl-*sn*-glycero-3-phosphoethanolamine
DOTAP: Cationic 1,2-dioleoyl-3-trimethylammonium-propane
DSPE-PEG2000-Mal: 1,2-Distearoyl-sn-glycero-3-phosphoethanolamine-N[maleimide (polyethylene glycol)-2000]
Egg PC: Egg L-α-phosphatidylcholine
EVs: Extracellular vesicles
FDA: Food and Drug Administration
HFNAP: Functionalized nucleic acid polymer
IV: Intravenous
LNPs: Lipid nanoparticles
MLGNPs: Multi-layer coated gold nanoparticles
MO: Monoolein
mRNA: Messenger RNA
PACEs: Poly(amine-co-ester)
PAMAM: Polyamidoamine
PBAE: Poly-beta-amino-ester
PDMAEMA: Polydimethylaminoethyl methacrylate
PEG: Polyethylene glycolylated lipid
PEG-DMG: 1,2-Dimyristoyl-rac-glycero-3-methylpolyoxyethylene
PEI: Polyethylenimine
PGD: Poly(glycolic acid)
PLA: Poly(lactic acid)
PLGA: Poly(lactic acid-glycolic acid)
PLL: Polylysine
PMOs: Phosphorodiamidate morpholino antisense-modified oligomers
PS-ODNs: Phosphorothioate oligonucleotides
RISC: RNA-induced silencing complex
SC: Subcutaneous
siRNA: Silencers RNA
SoyPC: L-α-phosphatidylcholine
Tf: Human holo-transferrin
TPGS: DL-α-Tocopherol methoxy-polyethylene glycol succinate
